# Diurnal and Nocturnal Behaviour of Cheetahs (*Acinonyx jubatus*) and Lions (*Panthera leo*) in Zoos

**DOI:** 10.3390/ani12182367

**Published:** 2022-09-11

**Authors:** Isabel Seyrling, Paul Wilhelm Dierkes, Anna Lena Burger

**Affiliations:** Bioscience Education and Zoo Biology, Goethe-University Frankfurt, 60438 Frankfurt, Germany

**Keywords:** activity budget, sleep, carnivores, zoo biology, animal behaviour, video observation

## Abstract

**Simple Summary:**

To date, few studies have examined both night-time and daytime behaviour of lions and cheetahs in zoos. To gain a deeper understanding of behavioural rhythms, this knowledge needs to be expanded. In our study, light was a strong influencing factor leading to different diurnal and nocturnal activity budgets. During the day, both species were significantly more active than during the night. These daily activity peaks correlated with feeding time. At night, lions showed increased resting behaviour with long sleep phases averaging more than 75 min per sleep event. Sleep phases are defined as minutes per sleep event with the animal’s body and head lying on the ground. The number of sleep phases, however, dropped slightly compared to the day. Cheetahs also had a higher length of sleep phases at night than during the day, although the increase was smaller than in lions. However, the number of sleep phases was significantly higher than during the day in all animals. Our results show that the behavioural rhythms of lions and cheetahs differ significantly during the day and night. The results show that studies that take into account 24-h rhythms are well suited to measure diurnal rhythms and, based on this, possibly derive statements on management and husbandry.

**Abstract:**

Mammals are constantly exposed to exogenous and endogenous influences that affect their behaviour and daily activity. Light and temperature, as well as anthropogenic factors such as husbandry routines, visitors, and feeding schedules are potential influences on animals in zoological gardens. In order to investigate the effects of some of these factors on animal behaviour, observational studies based on the analyses of activity budgets can be used. In this study, the daily and nightly activity budgets of six lions (*Panthera leo*) and five cheetahs (*Acinonyx jubatus*) from four EAZA institutions were investigated. Focused on the influencing factor light and feeding, we analysed these activity budgets descriptively. Behaviour was recorded and analysed during the winter months over an observation period of 14 days and 14 nights using infrared-sensitive cameras. Our results show that lions and cheetahs exhibit activity peaks at crepuscular and feeding times, regardless of husbandry. Thus, lions in captivity shift nocturnal behaviour familiar from the wild to crepuscular and diurnal times. In cheetahs, in contrast, captive and wild individuals show similar 24 h behavioural rhythms. The resting behaviour of both species is more pronounced at night, with cheetahs having a shorter overall sleep duration than lions. This study describes the results of the examined animals and is not predictive. Nevertheless, the results of this study make an important contribution to gaining knowledge about possible factors influencing the behaviour of lions and cheetahs in zoos and offer implications that could be useful for improving husbandry and management.

## 1. Introduction

Physiological processes such as sleep and wakefulness determine the circadian rhythm of an animal. However, these physiological processes are continuously influenced by external stimuli also known as zeitgeber [1]. Synchronising the inner clock to such zeitgebers as light and food availability is defined as entrainment [2]. In particular, the stimuli such as light and food are reported to influence activity and resting behaviour [1,3,4]. Based on these adaptations, animal behaviour can be divided into two levels with the animal either being physically active or resting [5,6]. Activity is crucial to life and requires more energy than resting [6]. Resting is seen as a state of regeneration in which the organism can gain new strength for the coming challenges of activity. Life is therefore a circadian sequence of rest and activity, oscillating daily (around 24 h), seasonally or circannually [1,7,8]. To better understand the biology and natural needs of a species, these rest-activity cycles have recently been increasingly studied in various mammals (for example, by [9,10,11,12]). The depiction of activity budgets of species to analyse circadian rhythms or deviation from those, are one of the most commonly used chronobiological method to improve animal welfare [13,14]. However, how these activity budgets are influenced by external variables is poorly understood compared to what is known about molecular and genetic mechanisms [15]. This finding can be attributed to methodological difficulties of activity measurements in free-living animals, without influencing the animals behaviour [16,17]. Behavioural observations, including the analysis and construction of activity budgets, can be used to fill these knowledge gaps [18,19].

Depending on their rest-activity cycle, mammal species can be classified as diurnal, nocturnal, cathemeral or crepuscular [20]. In the Felidae and Canidae, the main predators of ungulates, about 72% of the species extant are nocturnal [21]. Nevertheless, there are different activity patterns during the night among the Felidae, depending on their hunting strategies. Lions (*Panthera leo*), for example, usually hunt at night and therefore show a significantly higher nocturnal activity level than cheetahs (*Acinonyx jubatus*) which tend to hunt during dusk and dawn to avoid top predators [3,9,22]. Accordingly, cheetahs show more pronounced resting behaviour at night than lions [16]. Resting behaviour can be broken down into different stages of intensity, with sleep as the most intense form of rest. Sleep hereby is a state of immobility with greatly reduced responsiveness that is homeostatically regulated [23]. This behaviour state may also be defined by a specific body posture accompanied by arbitrary muscle twitches [23]. Various factors can lead to a change in the sensitive phases of sleep. Previous studies revealed that not only endogenous factors such as age and sex show an effect on the duration of sleep behaviour across phylogeny [16,24,25,26,27,28,29], but also exogenous factors seem to play a major role in the classification of sleep durations in mammals [15,30,31,32,33,34,35,36,37,38].

Diet has been shown to be one such exogenous factor [23,39]. Depending on the type of food, different sleep patterns can be identified: carnivores show the highest, omnivores a lower and herbivores the lowest sleep duration over 24 h [36,40]. In the field, hunting opportunities and the presence of competitors can influence predator activity [21,22]. Accordingly, feeding routines in zoos can also lead to circadian rhythm adjustment in mammals [4,41,42,43,44]. Captive felids therefore often spend the hours immediately before scheduled feeding times with increased activity [45]. In the wild, carnivores require more time and energy to search for, process and ingest food [45,46]. Furthermore, not only the feeding time, but also the feeding rate and the number of fasting days seem to have an impact on the resting and active behaviour of captive felids [43]. For example, lions in zoos have been reported to have lower activity levels on feeding days than on fasting days due to satiety [43]. Besides the availability of food of food [10,43], light [3,32,35] also has a decisive influence on the rest and activity behaviour of carnivores. In zoos, artificial light sources are often used in addition to natural light. This can lead to a temporal shift of the twilight and thus to a shift in the daily activity and rest behaviour [35,47]. Accordingly, the impact of light must be considered when analysing the activity budget of a captive species.

Different methods are used for time-resolved recording of diurnal behaviour in the field and in zoos. In the field, behaviour and movement recordings are often carried out with GPS collars often combined with motion sensors [48,49,50]. However, potential issues have to be considered when using these techniques, including sensor placement orientation and power supply [51]. In long-term studies, a high recording rate often cannot be guaranteed due to a weak energy supply [34,52]. In addition, animals need to be captured and immobilised in order to attach the sensors. Camera traps as non-invasive tools serve as a cheaper alternative to determine wildlife activity patterns in the field [22,53]. Moreover, camera traps can only provide information on the distribution of different animal species in a given area [54]. However, a continuous recording of an animal’s behaviour is not possible, as the camera traps are fixed and thus location-dependent [55]. As the behaviour of resting animals is more difficult to capture by this method, it is usually used to focus on activity patterns within a specific area of interest (e.g., water holes) [56,57,58]. Even though Breretron et al. [59] have shown that pinpoint sampling methods are very accurate, only continuous video recording allows time-resolved observation of wildlife, even during resting phases [59,60]. However, this observation method is of limited practicality in the wild, especially for recording nocturnal behaviour [61]. In contrast, zoos offer ideal opportunities to observe wildlife 24/7 via video recordings. The previously time-consuming analysis of video data will be simplified in the near future by suitable deep learning software solutions [62,63,64], so that longer periods and more individuals can be included in behavioural analyses. In a second step, the daily and nocturnal activity budgets for individual species can be drawn from these analyses.

The investigation of activity budgets of endangered species plays an important role in *ex-situ* management aspects [65]. As such, lions and cheetahs were classified as vulnerable by the IUCN in 2015 and 2016 [66,67]. Although both species are kept in many zoos, very little is known about their nocturnal resting behaviour, especially their sleep behaviour, and about the effect of feeding on resting behaviour. This study therefore investigated the diurnal and nocturnal behavioural rhythms of endangered lions and cheetahs. Particular attention focused on the influence of light and feeding on activity and resting behaviour over 24 h. Since changes in the animals’ welfare are often accompanied by changes in behaviour and circadian rhythms [14,18,68,69,70], results of this study can make an important contribution to a better understanding of the behavioural rhythms of lions and cheetahs in human care.

## 2. Materials and Methods

### 2.1. Data Collection and Behavioural States

Data for this study were collected during winter seasons 2019–2021 from five cheetahs and six lions housed at four EAZA (European Association of Zoos and Aquaria) institutions in Germany: cheetahs at Opel-Zoo Kronberg, Allwetterzoo Muenster and Cologne Zoo and lions at Zoo Neuwied, Cologne Zoo and Allwetterzoo Muenster. The age of the cheetahs varied between four and twelve years, that of the lions between four and twenty-two years (see Supplementary Table S1). In total, one female and four male cheetahs were studied as well as four female and two male lions. The behaviour was monitored and recorded via infrared-sensitive cameras (Lupus Geodome LE338) generating 1 frame per second with high-definition resolution. These camera settings were used for indoor and outdoor enclosures. The 24/7 recordings were carried out over a period of two weeks without interruption. Due to potential seasonal influences the recordings were only made during winter season. BORIS 7.7.3 software was used to analyse the video data [71]. The recorded data was analysed by applying continuous sampling for eight behaviour states (Table 1) [10]. Behaviour patterns were clustered into whether an animal was showing *active* or *rest behaviour.* The classification made for this purpose is based on a validated ethogram [10]. Continuous sampling allows for an accurate record of the behaviours with a time-resolved assignment of each individual behaviour pattern. The daytime recordings were defined from 7a.m.–7p.m., the night-time recordings from 7p.m.–7a.m. (during winter season). The recording and evaluation period refers to 14 days and nights per individual and was analysed to create a 24-h activity budget, based on the mean resting behaviour per second. The zookeepers filled out a detailed daily log to record special events, feeding times and the daily routine. In addition, the zookeepers completed a questionnaire on husbandry and management (e.g., enclosure size, food types and social relationship).

### 2.2. Data Analyses

To examine the diurnal and nocturnal activity budget of cheetahs and lions, behavioural data were aggregated per day (7a.m.–7p.m.) and night (7p.m.–7a.m.) for each individual. The Shapiro-Wilk normality test was used for normal distribution analyses. Afterwards *t*-test was used for unpaired samples, to test interspecific differences (between cheetahs and lions). In addition, a *t*-test was conducted to test for species-specific differences between day and night. To compare the different husbandry systems, depending on the normal distribution Kruskal–Wallis or Welch’s ANOVA were performed. For significant results, this was followed by post-hoc tests (Bonferroni test and Dunn–Bonferroni test). Additionally we made a descriptive analysis of the influence of the factors of light and feeding on the activity budgets. Statistical analyses and figures were performed in SPSS Version 27 (IBM, Armonk, NY, USA) and Microsoft Excel 365 (Redmond, Microsoft, WA, USA).

## 3. Results

**Figure 1:**
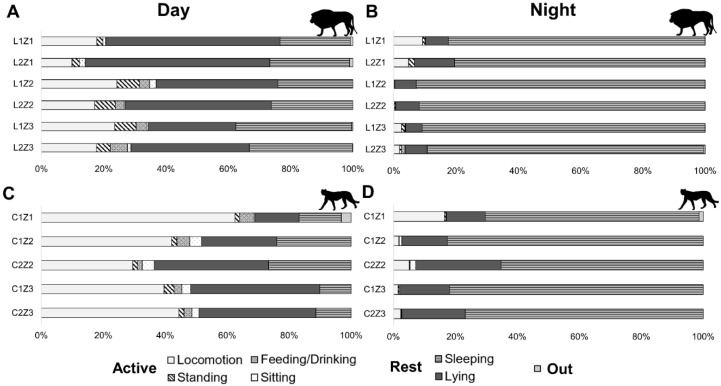
Diurnal (7a.m.–7p.m.) and nocturnal (7p.m.–7a.m.) activity budget across individuals and zoos: Percentages of behaviour states for lions (**A**,**B**) and cheetahs (**C**,**D**). The day phase is shown in the left column (**A**,**C**) and the night phase in the right column (**B**,**D**). For identification, individuals were categorised as follows: species/individual/zoo.

In this study, a total of 3696 h of video footage of cheetahs and lions from four different zoos was analysed. During the 14-day observation period, both cheetahs and lions spent significantly more time on active behaviours during the day than during the night (Table 2 and Figure 1). However, compared to lions, cheetahs showed significantly more active behaviour overall, both during the day (*t*-test: T = 21.2, *p* < 0.001, df = 27) and the night (*t*-test: T = 16.3, *p* = 0.041, df = 27). Cheetahs were locomoting about twice as much as lions during the day (cheetahs: 43.6%, lions: 18.4%). Interestingly, locomoting behaviour was nearly the same in both species during the night (cheetahs: 5.4%, lions: 3.1%). Not only locomotion but also the overall activity decreased significantly in cheetahs (6.4%) as well as in lions (4.2%) during the night. Accordingly, sleeping was the dominant behaviour pattern with cheetahs sleeping on average 75.1% of the night and lions 87.8%.

**Figure 2:**
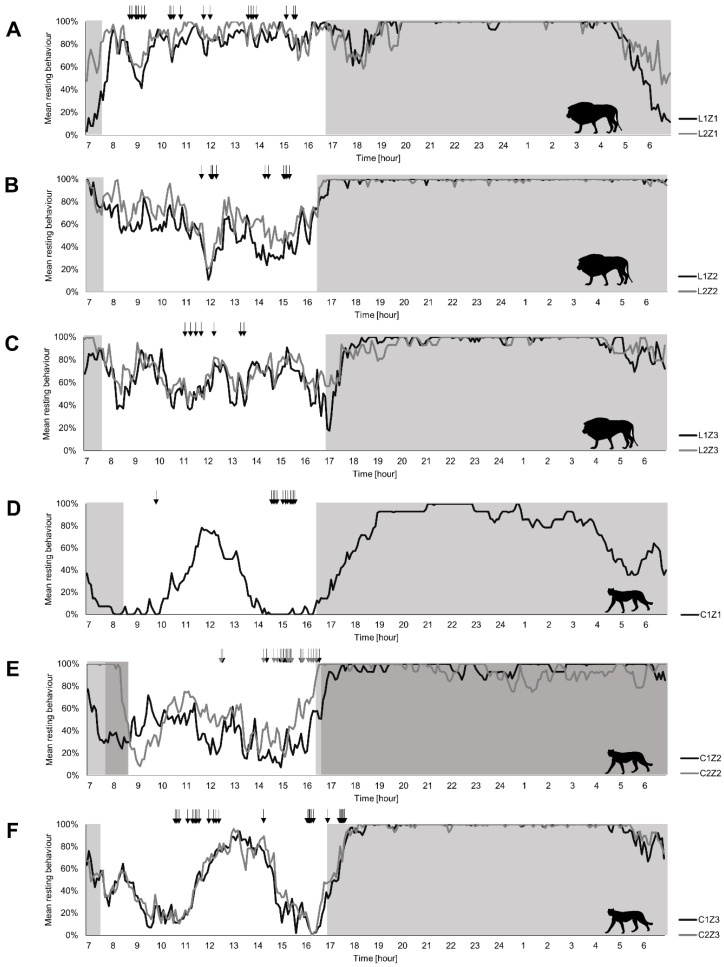
Time course of resting behaviour over 24 h: Resting behaviour of lions (**A**–**C**) and cheetahs (**D**–**F**) over a study period of 14 days (7a.m.–7p.m.) and nights (7p.m.–7a.m.). For identification purposes, individuals were categorised as follows: species/individual/zoo. Arrows mark feeding events throughout the observation period. Grey areas show dark periods. In zoo3 (**E**) dark periods vary between individuals due to different observation periods.

Our analyses of behavioural rhythms showed that light had an impact on the behaviour of lions and cheetahs in all zoos. With the onset of dusk, resting behaviour increased strongly and remained constant until the early morning hours (around 5 a.m.) (Figure 2). During the light period, the behaviour of both species changed more frequently than during the dark phase. However, during the dark phase, cheetahs switched more often between active and resting behaviour than lions did.

To investigate whether the behaviour of two individuals within an enclosure influences each other, they were descriptively compared (Figure 2A–C,F). The results show that the behavioural patterns of both individuals within one enclosure were strongly synchronised over the 24 h. In cases when two cats were kept together in the same enclosure, no differences in behavioural rhythmicity were observed between the two individuals.

Lions showed active behaviour mostly during diurnal and crepuscular hours, with a consistently high amount of resting behaviour during the night. A subsequent increase in activity was identified briefly before and after sunrise. During the day, lions showed various resting phases that were interrupted by feeding events (Figure 2A–C). Daily differences between zoos were tested with Welch’s ANOVA (Welch’s ANOVA: F = 29.06, df = 2, *p* < 0.001) followed by a post-hoc test with Bonferroni correction. Lions in zoo1 showed a significant lower amount of daily resting behaviour than lions in zoo2 (z1-z2day *p* < 0.001) and zoo3 (z1-z3day *p* < 0.001).

Nocturnal differences were tested with Kruskal–Wallis (Kruskal–Wallis: χ^2^ = 31.44, df = 2, *p* < 0.001) followed by a Dunn–Bonferroni post-hoc test. Lions in zoo1 showed a significant lower amount of nocturnal resting behaviour than lions in zoo2 (z1-z2night *p* < 0.001) and zoo 3 (z1-z3night *p* = 0.003) over 24 h; Furthermore, lions in zoo2 showed significant differences in the total time of resting behaviour during the night compared to zoo3 (z2-z3night *p* < 0.001).

As lions, cheetahs showed lower resting during the day and twilight. At night, in contrast, the cheetahs rested most of the time. Except for one cheetah (zoo1), the resting behaviours decreased again about one hour before sunrise.

Moreover, different diurnal and nocturnal activity levels have been observed depending on zoos for the cheetahs. Daily differences between zoos were tested with Welch’s ANOVA (Welch’s ANOVA: F = 33.51, df = 2, *p* < 0.001) followed by a post-hoc test with Bonferroni correction. The cheetah in zoo1 showed significantly more daily activity compared to cheetahs in zoo2 (z1-z2day *p* < 0.001) and zoo3 (z1-z3day *p* < 0.001). Nocturnal differences were tested with Kruskal–Wallis (Kruskal–Wallis: χ^2^ = 30.65, df = 2, *p* < 0.001) followed by a Dunn–Bonferroni post-hoc test. Cheetah in zoo1 showed significantly more nocturnal activity compared to cheetahs in zoo2 (z1-z2night *p* < 0.001) and zoo3 (z1-z3night *p* < 0.001). Despite the overall higher activity level of C1Z1, it showed a distinct resting phase at midday, similar to the cheetahs from zoo3 (Figure 2D–F). Cheetahs in zoo3 were fed irregularly throughout the day, whereas cheetahs in zoo1 and zoo2 got only food in the afternoon. From sunset onwards, all cheetahs showed an increasing resting behaviour, with high and constant proportions during the night, interrupted only by short periods of activity.

**Figure 3:**
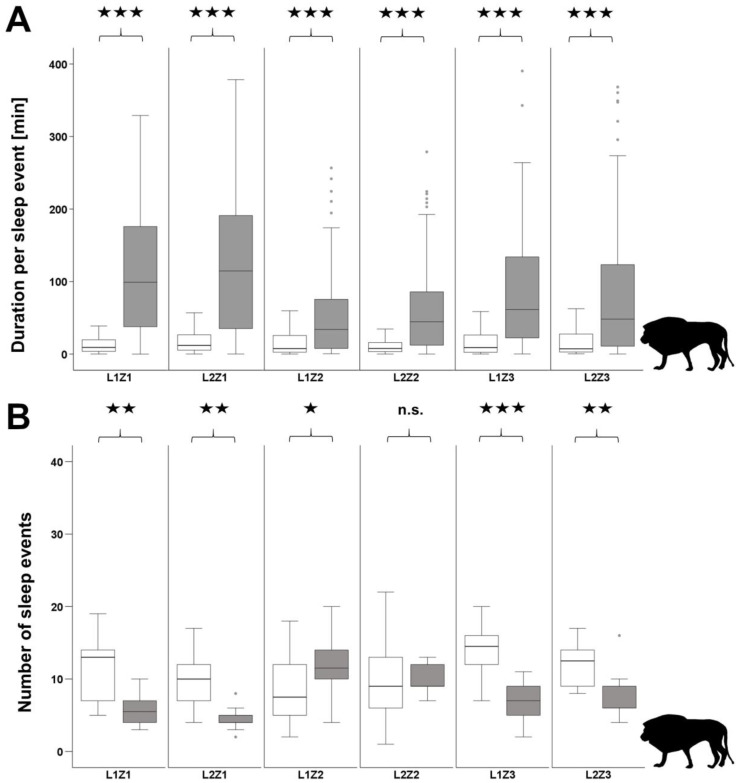
Diurnal (7 a.m.–7 p.m.) and nocturnal (7 p.m.–7 a.m.) sleep patterns of lions. (**A**) Duration per sleep event and (**B**) Number of sleep events per day (white boxes) and per night (grey boxes); n.s. > 0.05; ★ *p* < 0.05; ★★ *p* < 0.01; ★★★ *p* < 0.001) over the total observation period.

**Figure 4:**
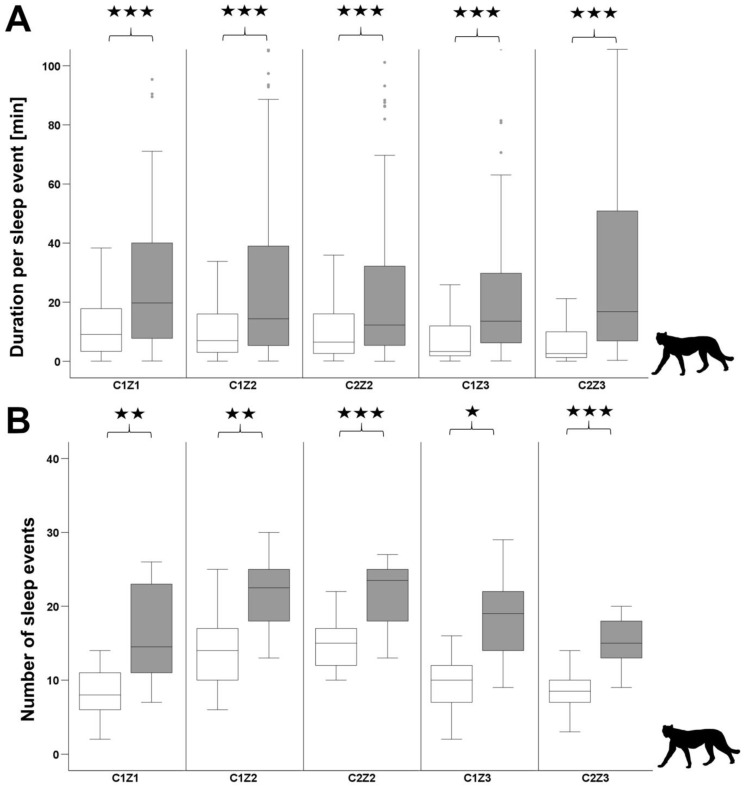
Diurnal (7 a.m.–7 p.m.) and nocturnal (7 p.m.–7 a.m.) sleep patterns of cheetahs. (**A**) Duration per sleep event and (**B**) Number of sleep events per day (white boxes) and per night (grey boxes); n.s. > 0.05; ★ *p* < 0.05; ★★ *p* < 0.01; ★★★ *p* < 0.001) over the total observation period.

The mean resting time over 24 h for lions was 21.1 ± 1.54 h and for cheetahs 18.36 ± 2.93 h. Significant differences between diurnal and nocturnal sleep behaviour were found for all individuals of the two species. In the lions, the length of sleep phases increases strongly during the night, whereas the number of sleep phases is significantly reduced in four individuals (Figure 3). Only one individual (L1Z2) showed an increase in the number of sleep phases and another individual (L2Z2) showed no change. The cheetahs showed a different picture. The length of sleep phases increased moderately but significantly in all individuals. In contrast to the lions, the number of sleep phases was significantly increased in all cheetahs (Figure 4).

Between both species, the daily duration per sleep event (*t*-test: T = 14.79, *p* < 0.001, df = 27) and the nocturnal duration per sleep event (*t*-test: T = 9.79, *p* < 0.001, df = 27) differed significantly. Lions spent more time per sleep event than cheetahs, both during the day and the night (Table 3). With regard to the number of sleep events, there were significant differences between the two species during the day and the night. Compared to lions, cheetahs showed significantly more sleep events during the day (*t*-test: T = 22.60, *p* < 0.001, df = 27) and the night (*t*-test: T = 10.76, *p* < 0.001, df = 27).

## 4. Discussion

### 4.1. Behavioural Rhythmicity over 24 Hours

This study presents a holistic 24-h activity budget of captive cheetahs and lions with special focus on their resting and sleep behaviour. Only the consistent observations over 24 h and many days and nights enabled us to determine a species-specific 24-h behavioural rhythm. So far, behavioural studies in zoos have mainly focused on the analysis of diurnal behaviour within narrow time windows [73,74,75]. Due to time constraints, scan sampling is a method commonly used to study stereotypic behaviour or the effect of enrichment [73,76,77,78]. This is often the case when analysing the daily moving behaviour of carnivores [73,79]. However, these studies do not check for a species’ behaviour profile over 24 hr. Even more, the analysis of behaviour observed only for a few hours a day might lead to misinterpretations such as generalisation, over- or underestimation [80,81]. In order to avoid misleading interpretation of observed behaviour patterns and rhythms and to study the influence of anthropogenic as well as individual biological factors on behaviour, the species-specific rhythm must be known first [30,82]. These 24-h activity budgets have to be based on continuous recordings around the clock and a second-by-second analysis [83].

Therefore, our study investigated the diurnal and nocturnal behavioural rhythms of captive lions and cheetahs and analysed potential influencing factors. Our results show a striking behavioural rhythm in lions and cheetahs over 24 h. During the day, periods of high activity were observed around feeding times. Later on, a further activity peak at dusk has been detected for both species. Similar to their wild conspecifics, captive lions also showed a high level of resting behaviour (72.8 ± 7.4%) during the day [3,84,85,86]. In contrast, the cheetahs in our study were found to rest only half of the day (48.2 ± 11.5%). These results are consistent with the daily activity budget observed in wild cheetahs [3,9,34].

### 4.2. Sleep Behaviour

Especially at night, we found inter- and intraspecific differences in nocturnal behavioural rhythms for the two species. Our results show cheetahs and lions mostly sleeping at night. However, we found large differences between lions and cheetahs regarding the number and length of sleep phases (Table 3). Lions slept most of the night, with sleep divided into few but long sleep phases. The results obtained on sleep behaviour are consistent with previous studies on captive lions [73,74,87]. In contrast to lions, cheetahs slept slightly less overall at night, with sleep divided into many short sleep phases. However, to our knowledge, so far there have been no studies on the nocturnal behaviour of captive cheetahs. Overall, we found some differences between the nocturnal behaviour of captive lions and their wild conspecifics. As wild carnivores are very difficult to observe, especially during the night, information on the sleep behaviour of wild cheetahs and lions is extremely rare. Most of the information on nocturnal behaviour has been gathered so far by using GPS-collars additionally equipped with accelerometers [50,88]. While wild lions are known to hunt during the night [85,86], captive lions were mostly inactive during the night [87]. This difference is most likely due to the fact that lions in zoos do not need to hunt for food and feeding in zoos takes place during the day [43,89,90]. Captive cheetahs interestingly show almost the same nocturnal behavioural rhythms as wild cheetahs [3,9,21,34]. In particular, the cheetahs in this study are active in the early morning hours (see Figure 2), which are used for hunting in the wild [91,92], although feeding does not occur at these times.

### 4.3. Food as Decisive Factor for the Behavioural Rhythm

In the wild, ecological factors such as activity of prey and predation risk potentially lead to different activity and hunting peaks between lions and cheetahs [93,94,95]. In zoos, obviously, hunting became obsolete for cheetahs and lions. However, in our study we determined feeding events to have a high impact on their behavioural rhythms. Various studies have already shown that feeding, access to food and hunger have an influence on the circadian rhythm [1,4,10,96]. In addition, an influence on this rhythm could be demonstrated in felids through carcass feeding [97]. Furthermore, fasting days with a low feeding frequency (1 time per week) may lead to higher activity levels in lions [46]. Individual differences between the animals of the different zoos could also be observed for this study. It is possible that these differences in daily activity and resting behaviour are due to different feeding times and numbers of feeding events [43]. To make further statements about this, the influence of the feeding schedule on the behaviour of these animals would have to be observed over a longer period than 14 days and nights. Consequently, feeding may have a strong influence on the activity budget of carnivores as shown by studies on leopards and lions [10,43,74]. Especially with scheduled feeding times, delayed feeding has been shown to result in a change in behaviour [41,44,98]. In our study, the daily activity patterns of the studied individuals were highly influenced by feeding routines. Animals anticipated scheduled feeding times and got active around these assumed periods of time. Accordingly, previous research also showed that lions in zoos exhibit activity peaks during the day around feeding times [43,74]. Nevertheless, lions also showed a high percentage of resting behaviour during the day which is in line with observations on the diurnal resting behaviour of their wild conspecifics [3,84,85,86]. In cheetahs, however, no fundamental differences to wild cheetahs could be found in the daily activity budget [9,22,32]. For example, wild cheetahs hunt mostly in the dawn and dusk hours [91,92] and feeding may extend beyond these time periods [32]. The cheetahs in this study are partially active during these periods.

### 4.4. Light and Season Shape Activity and Rest Behaviour

As already mentioned, the behavioural and physiological processes of animals are constantly influenced by endogenous and exogenous stimuli. Besides feeding, another stimulus is light [30,39]. As one of the strongest zeitgebers, light has a major impact on animal activity [1,27,70]. Results of our study confirm such an effect of light on behaviour by observing drastic differences in activity during the day and the night as well as during dusk. From dusk on lions and cheetahs were mainly resting and sleeping for the rest of the night. In the early morning hours, both species became more active again from two hours before sunset on. Besides light, the season has been shown to influence activity and resting behaviour of carnivores [34,99,100]. For instance, at high temperatures, main activity in cheetahs shifted from day to night [34]. Lions also adapted their activity and resting behaviour to the temperature by being more active on cooler days [100]. Compared to summer, in autumn months, lions were observed to be active later in the morning and earlier in the evening [86]. This leads to a natural variation in the day–night rhythm from summer to winter. Due to these potential seasonal influences and to ensure comparability between zoos, the study design was restricted to recordings during one season (winter).

## 5. Conclusions

This study presents a detailed 24-h activity budget of lions and cheetahs in zoos. The results show that their circadian rhythms are significantly influenced by external factors, which include light and feeding. Through 24/7 analysis, this study also provides insights into nocturnal behaviour, a time of day that has received less attention in behavioural studies [47]. The continuous behavioural recording offers ideal opportunities to make statements about biological rhythms, which can possibly be used in a next step to review and improve animal welfare or husbandry conditions [47]. In this context, zoos offer a unique potential for behavioural research. To conclude, the results of this study expand our knowledge of the 24-h behavioural rhythms of lions and cheetahs, highlight factors that influence them and enable for a further development of welfare assessments and management guidelines.

## Figures and Tables

**Table 1:** Ethogram of captive Felidae. The classification of behaviours into resting and activity was done according to Regaiolli et al. [72].

Behaviour State	Definition	Behaviour Image
**Active**		
Feeding/Drinking	Cat searches for food, ingests food by means of chewing or ingests water.	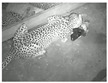
Locomotion	Walking, running or jumping with a forward locomotion.	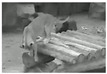
Standing	Cat is in an upright position, immobile, with all four paws on the ground and legs extended, supporting the body.	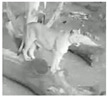
Sitting	Body is in an upright position, with the hind legs flexed and touching the ground, while front legs are extended and straight.	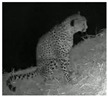
**Rest**		
Lying	Cat’s body is on the ground in a horizontal position, including on its side, back, belly or curled in a circular formation.	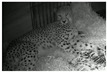
Sleeping	Cat is lying on the ground with its head down, performing minimal head or leg movement and is not easily disturbed.	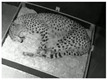
**Out**	The animal is not visible on records	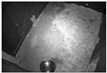

**Table 2:** Percentages of individual behaviour states of cheetahs and lions during day and night. Differences between day and night were tested for significance (right column).

	Lion (n = 6)					Cheetah (n = 5)				
Behaviour State	Day (%)	Night (%)	*p*-Value	T	df	Day (%)	Night (%)	*p*-Value	T	df
**Active**	26.8 ± 7.4	4.1 ± 3.4	<0.001	22.29	26	51.2 ± 11.5	6.3 ± 6.2	<0.001	38.38	26
Locomote	18.4 ± 4.8	3.1 ± 3.2	<0.001	18.11	26	43.6 ± 10.7	5.4 ± 5.7	<0.001	28.62	26
Standing	4.9 ± 2.1	0.8 ± 0.6	<0.001	14.38	26	1.9 ± 0.7	0.2 ± 0.1	<0.001	9.27	26
Feeding/Drinking	2.4 ± 1.9	0.2 ± 0.4	<0.001	4.78	26	3.1 ± 1.2	0.0 ± 0.0	<0.001	14.05	26
Sitting	1.1 ± 0.6	0.0 ± 0.0	<0.001	5.65	26	2.6 ± 1.3	0.7 ± 0.7	<0.001	7.92	26
**Rest**	72.8 ± 7.4	95.8 ± 3.4	<0.001	−22.29	26	48.2 ± 11.5	93.4 ± 6.2	<0.001	−38.24	26
Lying	44.6 ± 10.7	7.9 ± 2.3	<0.001	32.17	26	31.0 ± 10.2	18.3 ± 5.3	<0.001	10.37	26
Sleeping	28.2 ± 5.2	87.9 ± 4.7	<0.001	−43.30	26	17.2 ± 6.8	75.1 ± 6.9	<0.001	−50.55	26
**Out**	0.4 ± 0.4	0.1 ± 0.2	n.s.	3.16	26	0.6 ± 1.3	0.3 ± 0.5	n.s.	1.03	26

**Table 3:** Sleep behaviour of lions and cheetahs.

		Cheetah	Lion	*p*-Value	T	df
Total sleep time [hr]	Day	2.05 ± 0.23	3.38 ± 0.43	<0.001	7.481	27
	Night	9.01 ± 0.65	10.57 ± 1.5	<0.001	12.27	27
Sleep behaviour [%]	Day	17.1 ± 6.8	28.2 ± 5.2	<0.001	16.34	27
	Night	75.1 ± 6.9	87.9 ± 4.7	<0.001	59.04	27
Duration per sleep event [min]	Day	11.0 ± 13.8	18.6 ± 25.4	<0.001	14.79	27
	Night	28.6 ± 38.9	78.9 ± 87.7	<0.001	9.79	27
Sleep events (no.)	Day	11.2 ± 5.3	10.9 ± 4.6	<0.001	22.60	27
	Night	18.9 ± 5.7	8.0 ± 4.4	<0.001	10.76	27

## Data Availability

The data presented in this study are available on request from the corresponding author. The data are not publicly available due to the fact that the video recordings may contain pictures of zoo visitors or zoo staff whose consent for publication we do not have.

## Supplementary Materials

The following are available online at https://www.mdpi.com/article/10.3390/ani12182367/s1, Table S1: Metadata of analysed individuals.
