# Supplementary material for: Diurnal and Nocturnal Behaviour of Cheetahs (Acinonyx jubatus) and Lions (Panthera leo) in Zoos
Source: Animals (Basel). 2022 Sep 11;12(18):2367. doi: 10.3390/ani12182367 (PMC9495184; doi:10.3390/ani12182367)
Supplement: Supplementary file 1 [file animals-12-02367-s001.zip › animals-1868947-supplementary.pdf]

Table S1. Metadata of analysed individuals

ID	Species	Zoo	Age	Sex	Pride size (male:female)	Enclosure size (m²)
L1Z1	*Panthera leo*	Zoo1	6	F	1.1	800
L2Z1	*Panthera leo*	Zoo1	4	M	1.1	800
L1Z2	*Panthera leo*	Zoo2	13	M	1.1	536
L2Z2	*Panthera leo*	Zoo2	8	F	1.1	536
L1Z3	*Panthera leo*	Zoo3	13	F	0.2	750
L2Z3	*Panthera leo*	Zoo3	22	F	0.2	750
C1Z1	*Acinonyx jubatus*	Zoo1	6	M	1.0	688.5
C1Z2	*Acinonyx jubatus*	Zoo2	12	M	1.0	7500
C2Z2	*Acinonyx jubatus*	Zoo2	12	F	0.1	7500
C1Z3	*Acinonyx jubatus*	Zoo3	6	M	2.0	2000
C2Z3	*Acinonyx jubatus*	Zoo3	6	M	2.0	2000
